# Identification of grade and origin specific cell populations in serous epithelial ovarian cancer by single cell RNA-seq

**DOI:** 10.1371/journal.pone.0206785

**Published:** 2018-11-01

**Authors:** Andrew J. Shih, Andrew Menzin, Jill Whyte, John Lovecchio, Anthony Liew, Houman Khalili, Tawfiqul Bhuiya, Peter K. Gregersen, Annette T. Lee

**Affiliations:** 1 Robert S. Boas Center for Genomics and Human Genetics, The Feinstein Institute for Medical Research, Manhasset, New York, United States of America; 2 Donald and Barbara Zucker School of Medicine at Hofstra/Northwell, Hempstead, New York, United States of America; Cedars-Sinai Medical Center, UNITED STATES

## Abstract

Here we investigated different cell populations within ovarian cancer using single-cell RNA seq: fourteen samples from nine patients with differing grades (high grade, low grade and benign) as well as different origin sites (primary and metastatic tumor site, ovarian in origin and fallopian in origin). We were able to identify sixteen distinct cell populations with specific cells correlated to high grade tumors, low grade tumors, benign and one population unique to a patient with a breast cancer relapse. Furthermore the proportion of these populations changes from primary to metastatic in a shift from mainly epithelial cells to leukocytes with few cancer epithelial cells in the metastases. Differential gene expression shows myeloid lineage cells are the primary cell group expressing soluble factors in primary samples while fibroblasts do so in metastatic samples. The leukocytes that were captured did not seem to be suppressed through known pro-tumor cytokines from any of the cell populations. Single cell RNA-seq is necessary to de-tangle cellular heterogeneity for better understanding of ovarian cancer progression.

## Introduction

Ovarian cancer is the 5^th^ leading cause of cancer deaths in women living in the United States [1]. In 2017 alone, over 22,000 women were diagnosed with ovarian cancer and approximately 14,000 died from their disease. Epithelial ovarian cancers (EOCs) are broken down into four histological subgroups: serous, mucinous, endometroid and clear cell [2]. Serous ovarian cancers are the most common, comprising ~50% of cases [3] and can be further subdivided into high grade serous ovarian cancer (HGSOC) and low grade serous ovarian cancer (LGSOC) at ~90% and ~10% respectively [2].

The treatment options for HGSOC and LGSOC are similar with two standards of care, 1) Primary cytoreductive surgery (PCS) followed by adjuvant chemotherapy and 2) Neoadjuvant chemotherapy (NACT) followed by interval cytoreductive surgery (ICS) and adjuvant chemotherapy [4]. For both types of treatments, a combination chemotherapy consisting of platinum-based and taxane-based drugs (e.g. carboplatin and paclitaxel) is administered [5].

Each EOC type has a different prognosis with HGSOC having the worst outcome with the highest mortality rate of all the gynecological cancers. HGSOC is highly curable (>90% 5 year survival) if diagnosed early as local disease when the cancer is confined to the ovaries; however, most women are diagnosed with advanced stage metastatic disease. For these women, cure rates are exceptionally low: less than 25% of patients with late stage HGSOC will live more than 5 years [6]. Although HGSOC is initially sensitive to chemotherapy, almost invariably, relapse occurs followed by drug resistant progressive disease [7,8]. Overall 5-year survival of LGSOC is higher at 75% although this is dependent on having no residual disease following surgery as LGSOC has greater chemoresistance [9].

There has been no significant decrease in mortality rates in almost 30 years [10]. There is a critical need to improve our understanding of the underlying mechanisms leading to drug resistant ovarian cancer and identify potentially actionable therapeutic targets.

Ovarian cancer is a complex disease with significant tumor heterogeneity and as such there has been little success in identifying actionable targets. A meta-analysis of gene expression data from 1251 HGSOC tumors did not identify a collective prognostic gene expression signature [11]. Recently, Patch et al [12] performed a comprehensive whole genome analysis (DNA, RNA, miRNA, CNV, methylation) on 92 HGSOC tumors associated with different levels of drug response (i.e. refractory, resistant, or sensitive). Even with this in-depth analysis, no actionable targets or prognostic molecular profiles were identified. Given the rarity of LGSOC and its chemoresistance, most studies have been focused on recurrent disease using chemotherapy or hormone replacement therapy with mixed results [13]. Overall, analyses of bulk tumor tissue at the DNA and RNA levels have failed to provide results of significant clinical value.

Single-cell RNA-seq (scRNA-seq) allows for the quantitative and qualitative analysis of cell composition in complex tissues without *a priori* knowledge of the cell populations present. Several thousand genes can be quantitated simultaneously at the individual cell level. Using this approach we sought to identify commonalities and differences in cell composition of tumor samples from women with differing grades of serous epithelial ovarian cancer. Previous studies have examined ovarian cancer at the single cell level [14,15]; here we expand by assaying many more cells as well as identifying cell type specific differential expression.

## Materials and methods

### Subject recruitment, sample acquisition and sample processing

Women scheduled for surgery to evaluate a suspicious pelvic mass were recruited through the Tissue Donation Program at The Feinstein Institute. Pathological discard tissue, primary tumor and metastatic lesions (when available), were obtained at time of surgery and frozen for later analysis. Tissue was minced and frozen in 40% FBS, 40% RPMI and 20% DMSO. This freezing protocol routinely yields greater than 85% viable cells. This study was approved by the Institutional Review Board (IRB) of Northwell Health. See Table 1 for study subject characteristics.

**Table 1:** Patient ID with clinical information sample type obtained, number of cells captured per sample is in parentheses.

Patient ID	Patient Information						Sample IDs		
	Age at Diagnosis	Type of neoplasm	Stage	Previous HX of Breast Cancer	Neo-adjuvant	Race	Primary	Metastatic	Normal
BN1	56	Benign		No	No	White	BN1-P (223)		
HG1	70	HGSOC	IIIA	NA	No	--	HG1-P (252)	HG1-M (325)	
HG2F	67	HGSOC–Fallopian	IIIB	No	No	--	HG2F-P (260)	HG2F-M (259)	
HG3	66	HGSOC	IIIC	Yes	No	White	HG3-P (213)	HG3-M (312)	
HG4	54	HGSOC	IIIA	No	No	More than one race	HG4-P (174)		
HG5	69	HGSOC	IIIC	Yes	Yes	Black or African American	HG5-P (56)		
LG1	67	LGSOC	IA	No	No	White	LG1-P (194)		NM1 (344)
LG2	58	LGOSC	IIIC	No	No	Asian	LG2-P (130)	LG2-M (125)	
PN1	55	Peritoneal	IV	No	No	White	PN1-P (44)		

### Single-cell suspensions

Thawed tissue samples were digested and dissociated into single-cell suspensions using Miltenyi’s gentleMACS Octo dissociator and tumor dissociation kit following manufacturer’s instructions. Single cells were collected by straining digested tissue through a MACS Smart strainer (70um), washed, then layered over a Ficoll gradient to remove red blood cells and debris. To enrich the tumor cell fraction for live cells, dead cells were excluded using Dead Cell Removal kit and MS columns (Miltenyi Biotec) and remaining viable cells were prepared for scRNA-seq.

### Single-cell RNA-seq (scRNA-seq)

Using the BioRad droplet digital SEQ Single-cell Isolator and the Illumina SureCell Whole Transcriptome Analysis 3’ library prep kit, scRNA-seq was performed on isolated cells from ovarian tumor tissue samples following the manufacturer’s instructions. Briefly single-cells were encapsulated, lysed then barcoded within each droplet. Following first and second strand cDNA synthesis, Illumina’s Nextera technology was used to generate a library for NGS. Final libraries were assessed and quantified using a High Sensitivity DNA chip and a 2100 BioAnalyzer (Agilent Technologies) prior to sequencing on a NexSeq 500 high output flow cell.

### Data analysis

Sample data was demultiplexed and raw sequence files generated using bcl2fastq2 app in Illumina’s cloud application, Basespace, followed by the SureCell RNA Single-Cell application to extract the cell barcodes and the Unique Molecular Identifiers (UMIs). The raw sequence files were aligned to the hg19 human reference genome and gene expression was quantified. A knee plot of the genic UMIs per cell barcode was used to set thresholds for cells for downstream analysis. The data discussed in this publication have been deposited in NCBI’s Gene Expression Omnibus [16] and are accessible through GEO Series accession number GSE118828 (https://www.ncbi.nlm.nih.gov/geo/query/acc.cgi?acc=GSE118828).

The R [17] software package Seurat was used for further analysis [18,19]. Genes were initially filtered on expression in at least three cells and each cell needed to have at least 200 genes expressed. The entire dataset was then log-normalized with several factors regressed out: total number of UMIs and cell cycle scores. A principal component analysis of the most variable genes was performed and an elbow plot was used to select the principal components (PCs) capturing the most variance in the dataset. These PCs were used as edge weights in an unsupervised graph-based clustering to identify cell clusters. T-distributed stochastic neighbor embedding (tSNE) was used for visualization of the cell clusters. Expression levels of cell-type specific markers were used to determine the putative identities of each cell cluster. All colors used in plots were from Paul Tol’s color schemes and templates [20].

Once the cell clusters were identified, differential expression was done using a likelihood-ratio test tuned for single-cell expression, which assumes an underlying negative-binomial distribution as well as accounts for drop out using a zero-inflation regression. We defined differentially expressed genes as those with an adjusted p-value less than 0.05, an average log2 fold change greater than 1. For potential biomarkers we put further thresholds of being expressed in at least 50% of cells of the defining cluster with at least 50% difference of detectable expression of cells to the comparison group.

## Results

### Patient description

A total of 2911 cells were captured by scRNA-seq on nine patients with fourteen samples, see Table 1. All nine patients had a sample from the primary site, four had samples from a metastatic site and one had a sample from a normal ovary; all of the metastatic samples were from omentum proximal to the primary site. Five of the patients were diagnosed with HGSOC (with one being fallopian in origin), two were diagnosed with LGSOC, one had a cancer that was peritoneal in origin and one had a benign cystadenoma. Average age of diagnosis for the patients was 62.4 ± 6.5 years. All the tumors were diagnosed as stage III or greater with the exception of the benign cystadenoma and one of the LGSOC, which was at stage IA. Two of the patients (HG3 and NA1) had a previous history of breast cancer with one of them receiving neoadjuvant chemotherapy. The self-reported ethnicity had a breakdown of four White, one Asian, one Black/African-American, one with multiple races and two that did not self-report. Hematoxylin and eosin staining showed no visible lymphocyte infiltration in either tumor or metastatic lesions.

### Cell Identification

**Fig 1:**
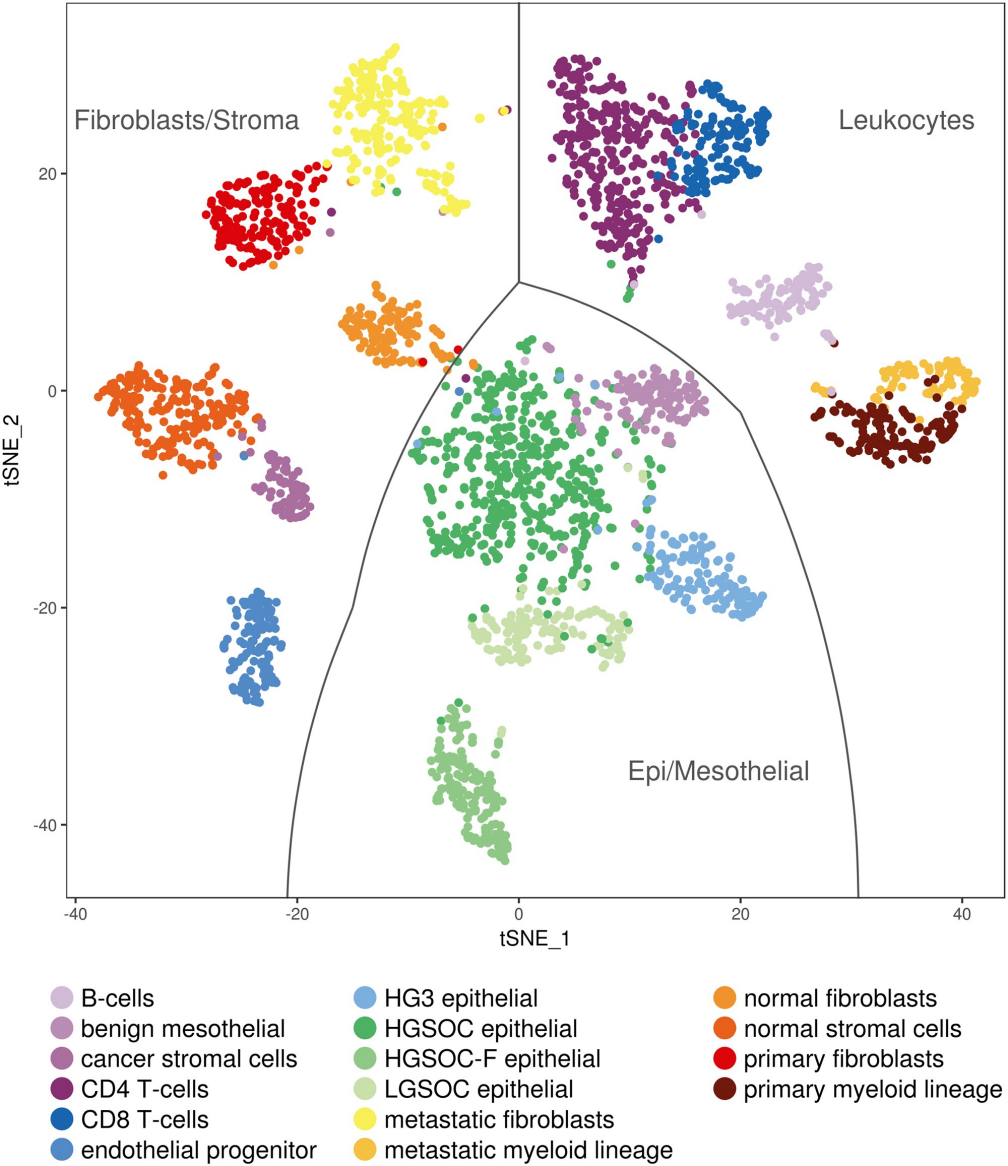
Identified cell clusters across nine patients with fourteen samples.

Pooled scRNA-seq data from all samples (tumor, normal, benign) identified sixteen distinct major clusters of cells that correlated with tumor origin, tumor status or known cellular gene expression patterns (Fig 1). The major clusters broadly divided into epithelial/mesothelial cells (EPCAM/CD326, KRT), lymphocytes (PTPRC/CD45, CD3E, CD19, MS4A1/CD20), endothelial cells (PECAM-1/CD31, CD34), fibroblasts (ACTA2, DCN, ACTB) and stromal cells (THY1/CD90, ENG/CD105, VIM, CD44), among others (S1 Fig). Pooled primary tumor data alone resulted in a large epithelial cell cluster that could be further subdivided into four groups, one group unique to HG3, one shared by HGSOC primary tumor samples, one shared by both LGSOC primary tumor samples and one cluster enriched in the ovarian tumor of fallopian origin HG2F. As expected normal ovary tissue displayed a significant presence of fibroblasts and stromal cells with no evidence of epithelial cells. ScRNA-seq data from a benign ovarian malignancy exhibited a unique cell cluster with epithelial markers, but is mesothelial in origin with other cell types divided among other clusters.

CD45 positive lymphocytes separated into five clusters with CD4+ and CD8+ T-cells (CD3D, CD4 and CD8, respectively), B-cells (CD19 and MS4A1/CD20 positive) and two clusters of myeloid lineage (CD14 positive). The myeloid clusters appeared to originate either from primary tumors or the metastatic lesion. Only the PECAM1/CD31 positive cluster of endothelial progenitor cells overlapped with all tissue samples analyzed.

### Cell population differences

**Fig 2:**
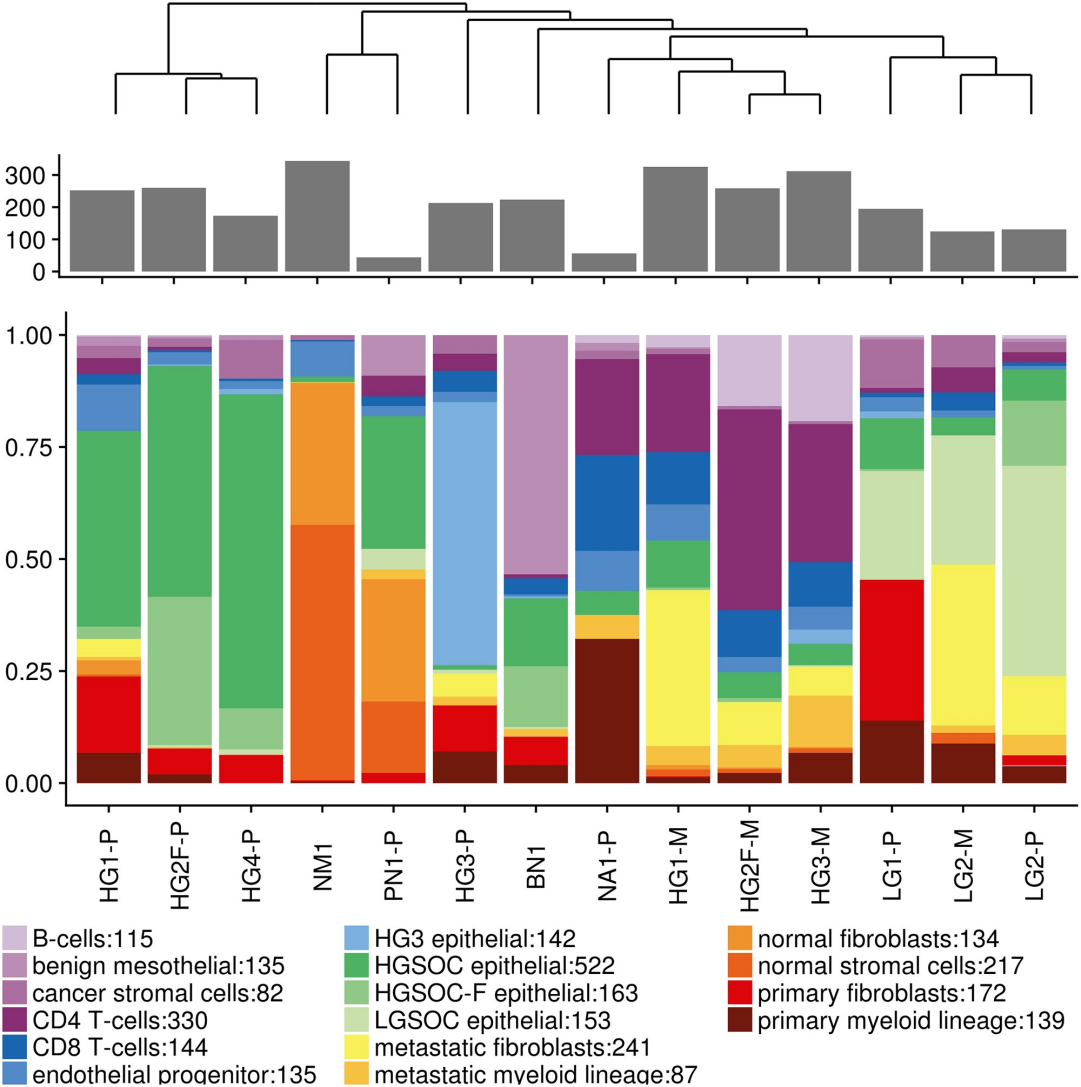
Proportions of cells making up each sample. Top is a dendrogram grouping similar samples by cell populations. Middle is the total number of cells captured. Bottom are bar plots of each cell type. Number following colon in legend is number of cells captured for each identified cell type.

Each subject sample had differing relative proportions of identified cells types, see Fig 2 for both percentages and numbers of cell types identified. An unsupervised hierarchical clustering of populations of cells from all subjects resulted in five groups. There were three major groups, which were predominantly from HGSOC primary tumor samples, HGSOC metastatic tumor samples and LGSOC samples. There were single outlier groups from benign, a primary tumor sample from HG3 and the normal and peritoneal samples clustering together. Surprisingly, the neoadjuvant treated primary tumor sample clustered with metastatic lesions. The outlier groups of benign and normal ovary samples were predictably comprised mostly of benign mesothelial cells as well as normal fibroblasts and stromal cells, respectively.

A definitive shift in cell populations between HGSOC primary tumors and corresponding HGSOC metastatic samples was observed (Fig 2). Primary tumor samples were predominantly epithelial cells (68.3% epithelial cells versus 11.1% lymphocytes), whereas corresponding metastatic samples were predominantly immune cells (66.2% lymphocytes versus 10.5% epithelial cells) with lesser amounts of epithelial cells, fibroblasts and stromal cells. Shifting from mainly epithelial to mainly lymphocytes seems to drive two of the big clusters (HGSOC primary and HGSOC metastatic) as well as the placement of the neoadjuvant treated sample in HGSOC metastatic: treatment killed off tumor epithelial cells. Clustering of LGSOC samples are based on the presence of LGSOC epithelial cells, which interestingly were present in a large quantity in the metastatic LGSOC sample. Two metastatic samples, HG2F-M and HG3-M, had significantly higher amounts of B-cells compared to all other samples (p-value < 2.2E-16 for both via Fisher’s test).

### Tumor epithelial cell expression

Using Seurat, potential biomarker discovery for all cancer epithelial cells versus every other cell type was performed (Table 2). Twelve differentially expressed genes were identified which included WFDC2, two members of the claudin family (CLDN3 and CLDN4) as well as three keratin genes (KRT8, KRT18, KRT19).

**Table 2:** Potential biomarkers comparing all four clusters of tumor epithelial cells versus all other cells.

Gene	Average Log2 Fold Change	Percentage in Cluster	Percentage out of Cluster	Adjusted p-value
All Tumor Epithelial Cells				
WFDC2	2.373	0.772	0.108	0
CLDN4	2.271	0.615	0.034	0
FXYD3	2.202	0.537	0.025	0
CD24	2.111	0.647	0.071	0
ELF3	2.061	0.636	0.061	0
CLDN3	2.045	0.623	0.031	0
MUC1	1.995	0.648	0.08	0
SPINT2	1.831	0.638	0.09	0
KRT8	1.767	0.664	0.086	0
SLPI	1.681	0.646	0.074	0
KRT18	1.640	0.671	0.134	0
KRT19	1.590	0.613	0.098	4.7E-297

Examining each tumor epithelial cluster versus the other three showed two clusters with potential biomarkers. HGSOC-F epithelial cells had unique identifiers with the top three expressed genes being TPPP3, C20orf85 and FOXJ1 (Table 3). The epithelial cell cluster unique to patient HG3 from this tumor had eleven differentially expressed genes with highest expression found in SST, TFF3 and PIGR. There were no distinct gene profiles for either of the HGSOC or LGSOC ovarian tumor epithelial cells, all highly expressed genes were also expressed within other epithelial clusters.

**Table 3:** Potential biomarkers of one cluster of tumor epithelial cells versus the other three, there were no specific biomarkers for either HGSOC or LGSOC epithelial cells.

Gene	Average Log2 Fold Change	Percentage in Cluster	Percentage out of Cluster	Adjusted p-value
HGSOC-F Epithelial				
TPPP3	3.377	0.885	0.083	1.1E-279
C20orf85	3.177	0.723	0.007	4.8E-299
FOXJ1	2.545	0.857	0.257	6E-159
RSPH1	2.455	0.615	0.033	1.2E-199
ZMYND10	2.285	0.569	0.02	4.7E-198
CAPS	2.197	0.731	0.218	2.3E-118
HG3 tumor epithelial				
SST	3.771	0.547	0.022	4.6E-181
TFF3	2.665	0.607	0.054	1.4E-176
PIGR	2.628	0.661	0.017	7.3E-243
GPNMB	2.010	0.65	0.021	4.7E-227
XBP1	1.824	0.768	0.173	6E-179
LYNX1	1.792	0.553	0.034	1.7E-169
AGR2	1.727	0.603	0.055	2.5E-170
DDIT4	1.639	0.846	0.272	2E-162
CXCL17	1.575	0.608	0.055	2.6E-173
KIAA1324	1.564	0.561	0.025	1.2E-180
NNMT	1.522	0.654	0.085	1.1E-164
STARD10	1.392	0.585	0.079	6.5E-142

### Non-epithelial cell expression

Comparison of normal fibroblasts and stromal cells to their tumor counterparts showed a striking upregulation of collagen genes including COL1A1, COL1A2, COL3A1, COL4A1, COL4A2 COL5A1, COL5A2, COL6A1 and COL6A3 as well as collagen support genes, SPARC, SERPINH1 and SERPINE1 (Table 4). COL4A1, COL4A2, SPARC, SERPINH1, and SERPINE1 were also expressed within the endothelial progenitor cluster. Cancer fibroblast and stromal cells had increased expression of MMP and MMP-related genes: MMP2, MMP11 and TIMP1. While many secreted factors were expressed by myeloid lineage cells (primary and metastatic), metastatic fibroblasts and cancer stromal cells, as expected relatively few were observed in tumor epithelial cells (S1 Table).

**Table 4:** Collagen genes and matrix metalloprotease genes upregulated in tumor fibroblasts and stromal cells versus normal.

Gene	Average Log2 Fold Change	Percentage in Cluster	Percentage out of Cluster	Adjusted p-value
COL1A2	3.416	0.746	0.139	2.8E-84
COL3A1	3.093	0.702	0.097	1.88E-77
COL1A1	3.481	0.733	0.231	1.17E-71
COL6A3	1.985	0.567	0.083	1.09E-50
COL5A1	1.825	0.509	0.05	7.49E-46
COL5A2	1.634	0.488	0.028	4.77E-45
COL4A1	2.313	0.542	0.094	4.18E-42
COL4A2	1.871	0.554	0.122	3.91E-39
COL6A1	1.154	0.676	0.317	4.17E-30
MMP2	1.152	0.377	0.047	1.27E-26
TIMP1	1.457	0.718	0.425	2.08E-25
MMP11	1.185	0.189	0.008	1.03E-12

Doing a differential expression between the two clusters of cancer fibroblasts, we found metastatic fibroblasts were found to express higher levels of soluble factors compared to primary fibroblasts including, CXCL12, S100A6, S100A10, SFRP2,SFRP4,IGF1, CXCL14, ANGPTL4 and IL6 (Table 5). Metastatic fibroblasts also had increased expression of two complement cascade genes C3, CFB and an inhibitor of C1 gene, SERPING1. Tumor fibroblasts had increased expression of C7. Comparison between the primary tumor myeloid lineage cells and the metastatic myeloid lineage cells showed elevated levels of CC2, CC3, CC4, CXCL8 and TNF in the primary tumor.

**Table 5:** Comparison of secreted factors and complement system genes in primary vs metastatic myeloid lineage and primary vs metastatic fibroblasts.

Gene	Average Log2 Fold Change	Percentage in Cluster	Percentage out of Cluster	Adjusted p-value
Secreted factors in primary myeloid not in metastatic				
TNF	1.088	0.503	0.287	1.41E-02
CXCL8	1.310	0.621	0.387	1.13E-04
CCL2	1.371	0.26	0.039	8.68E-05
CCL3	1.465	0.774	0.436	1.08E-10
CCL4	1.575	0.746	0.387	6.29E-12
Secreted Factors in metastatic fibroblasts not in primary				
SFRP2	2.078	0.023	0.36	3.32E-25
S100A10	1.892	0.168	0.709	4.59E-44
CXCL12	1.853	0.137	0.63	1.80E-46
SFRP4	1.685	0.275	0.421	7.32E-12
S100A6	1.538	0.519	0.87	4.54E-46
IGF1	1.436	0.111	0.438	4.75E-18
CXCL14	1.353	0.099	0.26	1.66E-07
ANGPTL4	1.254	0.065	0.236	4.83E-11
IL6	1.115	0.057	0.202	1.09E-04
Complement System in primary fibroblasts not in metastatic				
C7	1.239	0.79	0.195	3.45E-16
Complement System in metastatic fibroblasts not in primary				
C3	2.823	0.08	0.849	8.68E-101
CFB	2.102	0.076	0.449	4.80E-31
SERPING1	1.469	0.443	0.822	6.05E-44

### Comparison of cell populations to TCGA HGSOC sub-types

The Cancer Genome Atlas (TCGA) collected 489 HGSOC samples and performed mRNA expression, microRNA expression, promoter methylation and DNA copy number assays [21]. Clustering of the samples based on both mRNA and microRNA expression showed four stable subtypes within HGSOC that were labeled differentiated, immunoreactive, mesenchymal and proliferative.

Using genes sets defining the four HGSOC subtypes, we used the average expression of groups of cells to classify samples, first by patient sample ID then by identified cell clusters. The threshold of classification was defined as two standard deviations above the mean of the combined group comparisons. By patient sample ID, only two patients were identified with HGSOC sub-types (Table 6), LG2-P as differentiated and LG2-M as mesenchymal, which are LGSOC samples.

**Table 6:** Comparison of gene expression of TCGA ovarian cancer sub-types by patient samples (top) and cell clusters (bottom).

	Differentiated	Immuno-responsive	Mesenchyaml	Proliferative
BN1-P	3.246	0.712	1.062	0.189
HG1-P	1.144	0.613	1.476	0.544
HG1-M	1.113	0.887	2.299	0.347
HG2F-P	1.942	0.240	0.657	0.608
HG2F-M	1.351	1.231	0.574	0.345
HG3-P	2.062	1.037	1.426	0.464
HG3-M	1.498	1.681	0.956	0.350
HG4-P	1.523	2.527	0.732	0.366
HG4-M	1.934	0.228	0.834	0.292
LG1-P	0.999	0.590	1.902	0.395
LG1-N	0.871	0.124	1.973	0.416
LG2-P	3.604	0.514	1.449	0.284
LG2-M	2.968	0.684	3.831	0.236
PN1-P	1.594	0.291	1.635	0.690
B-cells	0.194	1.496	0.318	0.442
benign epithelial	4.216	0.621	0.654	0.180
cancer stromal cells	0.447	0.142	4.559	0.355
CD4 T-cells	1.848	1.245	0.353	0.331
CD8 T-cells	1.105	1.594	0.568	0.349
endothelial progenitor	1.112	0.208	2.012	0.481
HGSOC-F tumor epithelial	2.946	0.202	0.347	0.424
LGSOC epithelial	3.808	0.227	0.488	0.304
metastatic fibroblasts	1.304	0.270	5.074	0.389
metastatic myeloid lineage	1.129	3.931	1.088	0.261
normal fibroblasts	0.553	0.159	1.429	0.616
normal stromal cells	0.937	0.076	2.439	0.293
HGSOC epithelial	1.691	0.226	0.413	0.569
primary fibroblasts	0.493	0.174	3.337	0.582
primary myeloid lineage	0.744	3.732	1.354	0.272
HG3 tumor epithelial	2.753	0.822	0.711	0.491

Those highlighted in green are identified as that specific sub-type.

However using identified cell clusters, there was significantly more correlation with the prescribed function of each sub-type (Table 6). Benign epithelial cells and LGSOC epithelial cells were identified as the differentiated subtype. Primary and metastatic myeloid lineage cells showed increased expression of the immunoresponsive subtype. Primary fibroblasts, metastatic fibroblasts and cancer stromal cells were identified as the mesenchymal subtype while normal fibroblasts and normal stromal cells had no similarities to any of the HGSOC subtypes. None of the clusters showed enrichment of proliferative subtype. Here we see that myeloid lineage defines immunoresponsive subtype, while tumor fibroblasts and stromal cells define mesenchymal, and epithelial cells define differentiated. The proliferative subtype may either be a cancer type or a rare cell population that was not captured in this study.

## Discussion

With scRNA-seq, we can investigate intra and inter tumor heterogeneity gene expression differences at cellular resolution. Given that epithelial cells are the predominant dysregulated cell type in epithelial ovarian cancer, we first wanted to compare these cells to all others and their potential relationship to cancer progression. The genes we identified at the single cell level in all cancer epithelial cells are concordant with previous bulk RNA seq studies. WFDC2 has been shown to be a biomarker for ovarian cancer [22] and overexpression promotes ovarian tumor growth [23]. Similarly, SCGB2A1 is expressed in all ovarian cancers [24]. CD24 has prognostic value in survival as a marker for cancer stem cells [25–28] whereas CLDN3 and CLDN4 have been shown to regulate the epithelial to mesenchymal transition [29]. These expression profiles imply that in ovarian cancer, epithelial cells are transitioning into a mesenchymal state, which is a hallmark of cancer.

Although all the malignant tumors had been classified as serous ovarian cancer, there are specific subsets of cancerous epithelial cells that correlate with pathological findings. Specific expression patterns of subsets of tumor epithelial cells expressed functional genes related to their origin. HGSOC-F epithelial cells biomarker TPPP3 is a tubulin polymerization protein that has been previously implicated in colorectal cancer [30], while the other three genes are necessary for proper cilia function. FOXJ1 is a differentiating factor for ciliated epithelial cells in the neonatal oviduct [31], RSPH1 is necessary for proper cilia function [32] and ZMYND10 affects dynenin motor function in cilia [33]. Given that these cells are fallopian in origin, their expression of cilia related genes is not unexpected as cilia are necessary in normal fallopian function. However these genes may also provide extra motility and transitioning to a mesenchymal state for the subtype of HGSOC that is fallopian in origin.

Two genes identified in HG3 tumor epithelial cells are broadly implicated in many cancers with worse prognoses, PIGR [34,35] and AGR2 [36,37]. They are the only cells in this dataset that express two soluble factors, a cytokine CXCL17 which has been found to promote tumor progression in other cancers [38–40] and a hormone SST, which suppresses other growth factors and has been considered as a potential pharmaceutical target in cancers [41,42]. HG3 is the only patient that had a previous cancer diagnosis without neoadjuvant chemotherapy: a breast malignancy more than ten years prior to her ovarian cancer diagnosis. Whether this cancer is a late recurrence of breast cancer or different in context of increased cancer risk, the biomarkers here imply a more aggressive phenotype with different activating factors.

The idea of pre-metastatic niche was postulated by Stephen Paget in 1889 as a tumor “seed” finding its appropriate “soil” [43]. Formation of a pre-metastatic niche involves tumor associated cells to create a hospitable microenvironment for tumor cells through specific secreted factors [44]. Secreted factors were mainly being produced by cells of myeloid lineage and metastatic fibroblasts especially compared to epithelial cells in primary tumor samples. Given there were few epithelial cells in metastatic samples it is unclear if they are producing many secreted factors. Therefore myeloid lineage cells are crucial in both primary and metastatic ovarian tumors, with fibroblasts playing a more important role in metastatic growth than in primary.

Collagen has a complex role in cancer progression. Initially thought to provide a barrier to tumor invasion, collagen has been shown to remodel the ECM to help promote angiogenesis, tumor invasion and migration [45]. Furthermore, matrix metalloproteases work to degrade the ECM to increase tumor invasion, particularly in metastasis [46]. Increased expression of both types of genes in ECM remodeling is thought to play a role in fibrosis/desmoplasia that is seen in many types of cancer [47]. We saw significant desmoplasia in pathology slides that was increased in metastatic samples compared to primary samples. High expression of several collagens and MMP-related genes in our dataset was primarily in cancer fibroblasts and stromal cells, not directly from the cancer epithelial cells themselves. This supports the theory that tumor stroma plays an important role in the tumor development [48]. In addition, there was high expression of COL4A1, COL4A2, SERPINH1 and SPARC in endothelial cells. Type IV-collagen has previously been shown to be necessary in angiogenic functions of endothelial cells [49,50], with SERPINE1 implicated in maintaining ECM of arterial walls [51]. SPARC and SERPINH1 play important roles in type-IV collagen synthesis and function [52]. Concurrent expression of these genes in four different clusters of cells imply collagen IV is crucial in the recruitment of endothelial cells for angiogenesis. Furthermore, as endothelial cells are the only cluster of cells that are represented in normal, primary tumor and metastatic tumor this implies there is no cell or class state switch in endothelial cells and these cells are functioning normally given their microenvironment and stimuli.

Creating a pro-tumor environment also requires avoiding immune surveillance from B-cells and T-cells, which can have both negative regulation and positive regulation of cancer, depending on specific tumor microenvironment (B-cells reviewed in [53], T-cells reviewed in [54]). Regulatory B-cells are expected to promote tumor growth by suppressing immune response through IL-10 and STAT3. Our cluster of B-cells do not seem to be regulatory B-cells, as they are CD38- and CD27- and with most similar marker expression as mature naive. More importantly, they do not express any IL-10 or STAT3. We also only see minor sporadic expression of markers for T-reg cells (PDCD1/PD-1, FOXP3), with little expression of pro-tumor cytokines, IL-10 and CCL22. Presence of immune cells in metastatic tumors is paradoxical, especially in light of no expression of pro-tumor factors. In fact we actually see expression of interferon-Ɣ in CD8 cells which is thought to by anti-tumor [55,56]. Recent research has shown a pro-tumor role of interferon-Ɣ through expression of PD-L1 [57], but expression of PD-L1 is not evident in our dataset.

One critical component of managing the immune system is the complement system, which regulates the innate immune system and plays a role in tumor progression [58,59]. Metastatic fibroblast cells overexpress C3, which is one of the key components that must be activated in the complement system. In murine models C3 has roles in tumor growth [60] and angiogenesis [61]. Furthermore, ascitic fluid taken from ovarian cancer patients showed surface deposition of C3 with increased CFH (which is only mildly overexpressed in these cells in our dataset) [62,63]. The other genes overexpressed in these cells are inhibitors, CFB and SERPING1. Adding to this, metastatic fibroblasts are also the only cells expressing CXCL12, which along with CXCR4 play a pivotal role across many cancers [64–66], including ovarian cancer [67,68]. We see CXCR4 expressed only in B-cells and T-cells. Metastatic fibroblasts also express two S100 family proteins, S100A10 and S100A6, which are important in creating inflammation for tumor growth and metastases [69,70]. Taken together it appears that metastatic fibroblasts are creating an inflammatory environment through CXCL12 and S100 family proteins while suppressing immune response through complement system inactivation.

## Conclusions

Our results show four major malignant epithelial cell types seen in nine patients diagnosed with either high grade or low grade serous epithelial ovarian cancer. Gene profiles were found for all four in aggregate or for two clusters individually, particularly HGSOC that is fallopian in origin and one in a patient with breast cancer recurrence. These may serve either as surveillance option, e.g. CA125 in ovarian cancer [71] or treatment, e.g. HER2 inhibitors in breast cancer [72,73]. Secondly, we found that primary tumor epithelial cells secrete much less factors in comparison to cells of myeloid lineage or fibroblasts. Between primary and metastatic tumors, fibroblasts secrete increasing levels of necessary factors to fuel metastatic growth, with the caveat that we captured only a few metastatic epithelial cells and may see more secreted factors with a larger sample size. Metastatic fibroblasts could potentially be tumor epithelial cells that have fully transformed (as many of the tumor epithelial expressed genes imply), but metastatic fibroblasts we observed do not express PROM1/CD133, a well described cancer stem cell marker [74]. Third, once ovarian cancer metastasizes, relatively few tumor cells are required to create and maintain a favorable microenvironment as evidenced by the shift of cell populations from epithelial cells to lymphocytes. Fourth, B-cells and T-cells do not appear to be suppressed through known pro-tumor cytokines, but could be suppressed indirectly through complement pathways. Finally, using previous data from ovarian cancers analyzed by TCGA, we find cancer subtype to be correlated with specific cell types.

Only through high-resolution studies at single cell resolution are we able to identify and quantify heterogeneity within tumors. Our ability to study patient derived primary and corresponding metastatic lesions using high-throughput single cell analysis represents a unique opportunity to study ovarian cancer without *a priori* knowledge of tumor and stromal cell inter-relationships. Thus single cell assessment of patient samples can provide critical information needed to understand ovarian cancer progression.

## Supporting information

S1 Fig Coloring of tSNE plot by known markers defining each cluster of cells. (TIFF)

S1 Table Gene expression of secreted factors increased in each cluster. (XLS)

S2 Table All genes upregulated in each cluster. (XLS)
