# Correction: Identification of grade and origin specific cell populations in serous epithelial ovarian cancer by single cell RNA-seq

**DOI:** 10.1371/journal.pone.0208778

**Published:** 2018-12-04

**Authors:** Andrew J. Shih, Andrew Menzin, Jill Whyte, John Lovecchio, Anthony Liew, Houman Khalili, Tawfiqul Bhuiya, Peter K. Gregersen, Annette T. Lee

The following information is missing from the Funding section: This study was funded by Partners Council for Women's Health.
