# Supplementary figures and images for: Identification of grade and origin specific cell populations in serous epithelial ovarian cancer by single cell RNA-seq
Source: PLoS One. 2018 Nov 1;13(11):e0206785. doi: 10.1371/journal.pone.0206785 (PMC6211742; doi:10.1371/journal.pone.0206785)

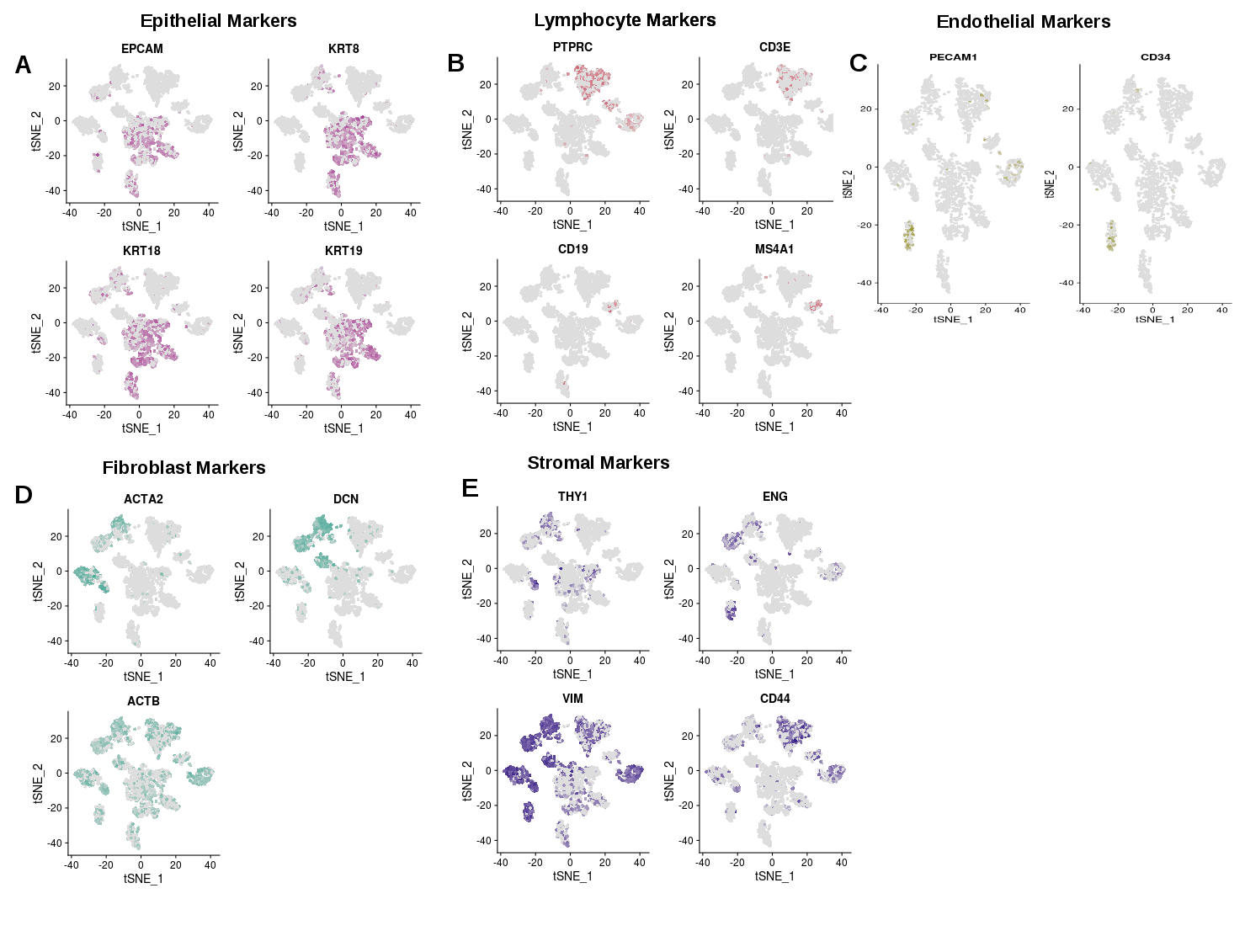

Supplement: S1 Fig — (TIFF) [file pone.0206785.s001.tiff]
